# Mammography features for early markers of aggressive breast cancer subtypes and tumor characteristics: A population‐based cohort study

**DOI:** 10.1002/ijc.33309

**Published:** 2020-10-06

**Authors:** Pui San Tan, Maya Alsheh Ali, Mikael Eriksson, Per Hall, Keith Humphreys, Kamila Czene

**Affiliations:** ^1^ Department of Medical Epidemiology and Biostatistics Karolinska Institute Solna Sweden; ^2^ Swedish eScience Research Centre (SeRC) Karolinska Institute Stockholm Sweden; ^3^ Department of Oncology Södersjukhuset Stockholm Sweden

**Keywords:** breast cancer, mammography, microcalcification, BIRADS, density change

## Abstract

Current breast cancer risk models identify mostly less aggressive tumors, although only women developing fatal breast cancer will greatly benefit from early identification. Here, we evaluated the use of mammography features (microcalcification clusters, computer‐generated Breast Imaging Reporting and Data System [cBIRADS] density and lack of breast density reduction) as early markers of aggressive subtypes and tumor characteristics. Mammograms were retrieved from a population‐based cohort of women that were diagnosed with breast cancer from 2001 to 2008 in Stockholm‐Gotland County, Sweden. Tumor and patient characteristics were obtained from Stockholm Breast Cancer Quality Register and the Swedish Cancer Registry. Multinomial logistic regression was used to individually model each mammographic feature as a function of molecular subtypes, tumor characteristics and detection mode. A total of 4546 women with invasive breast cancer were included in the study. Women with microcalcification clusters in the affected breast were more likely to have human epidermal growth factor receptor 2 subtype (odds ratio [OR] 1.78; 95% confidence interval [CI] 1.24‐2.54) and potentially less likely to have basal subtype (OR 0.54; 0.30‐0.96) compared to Luminal A subtype. High mammographic cBIRADS showed association with larger tumor size and interval vs screen‐detected cancers. Lack of density reduction was associated with interval vs screen‐detected cancers (OR 1.43; 1.11‐1.83) and potentially of Luminal B subtype vs Luminal A subtype (OR 1.76; 1.04‐2.99). In conclusion, microcalcification clusters, cBIRADS density and lack of breast density reduction could serve as early markers of particular subtypes and tumor characteristics of breast cancer. This information has the potential to be integrated into risk models to identify women at risk for developing aggressive breast cancer in need of supplemental screening.

## What's new?

Current breast cancer risk models do not specifically identify women at risk of aggressive breast cancer. Using mammograms from a population‐based cohort of women diagnosed with invasive breast cancer, this study found that women with microcalcification clusters in the affected breast were more likely to have the HER‐2 subtype and potentially less likely to have the basal subtype compared to Luminal‐A subtype. Women with no breast density reduction had increased probability of interval versus screen‐detected cancer and possibly of Luminal‐B versus Luminal‐A subtype. These mammography features could potentially serve as early markers for aggressive breast cancer subtypes and tumor characteristics.

**Abbreviations**
BIRADS: Breast Imaging Reporting and Data System
BMI: body mass index
cBIRADS: computer‐generated Breast Imaging Reporting and Data System
CI: confidence intervals
ER: estrogen receptor
FISH: fluorescence in situ hybridization
HER‐2: human epidermal growth factor receptor 2
HRT: hormone replacement therapy
OR: odds ratio
*P*: *P*value
PR: progesterone receptor

## 1 INTRODUCTION

Breast cancer affects around 2 million individuals a year globally.^1^ Better treatments and intensified screening are reflected in continuous improvements of prognosis.^2^ Most screening programs use a one‐size‐fits‐all approach, which means that women are screened at regular intervals at certain ages.^3^ Several risk models have been developed to enable identification of women at high risk and thereby in need of additional examination procedures.^4^, ^5^, ^6^ Current breast cancer risk models do not specifically identify women at risk for aggressive breast cancer, which is a drawback since only women potentially diagnosed with a fatal breast cancer will greatly benefit from screening.^4^, ^5^, ^6^, ^7^, ^8^, ^9^

To date, there remains a lack of early markers for potentially aggressive breast cancer from routine mammograms. Microcalcifications commonly found on mammographic screenings are routinely used for diagnosis of early breast cancer and in particular ductal carcinoma in situ.^10^ They are formed in breast tissues through physiological mineralization processes of calcium.^11^ Recent studies have suggested that the presence of microcalcifications might increase the likelihood for particular subtypes of breast cancer, for example, the human epidermal growth factor receptor 2 (HER‐2) subtype.^12^, ^13^

Furthermore, while earlier studies have found that mammographic density could be associated with aggressive breast cancer subtypes and tumor characteristics including interval breast cancer,^14^, ^15^, ^16^, ^17^, ^18^, ^19^ it remains unclear of the utility of mammographic density when measured in the clinical context using the computer‐generated Breast Imaging Reporting and Data System (cBIRADS).^20^ In addition, emerging studies are showing that women who do not experience physiological mammographic density reduction with age might have an increased risk of breast cancer.^21^, ^22^, ^23^ However, little is known regarding the association of mammographic density change with molecular subtype and tumor characteristics.

In our study, we evaluated the associations of mammographic features (microcalcification clusters, cBIRADS density^20^ and density change) with specific molecular subtypes of breast cancer, tumor characteristics and mode of detection. Potentially, the integration of mammography features into existing risk models, particularly for specific tumor subtypes, will allow the identification of women at risk of developing aggressive breast cancer in need of supplemental screening.

## 2 METHODS

### 2.1 Study population

**FIGURE 1:**
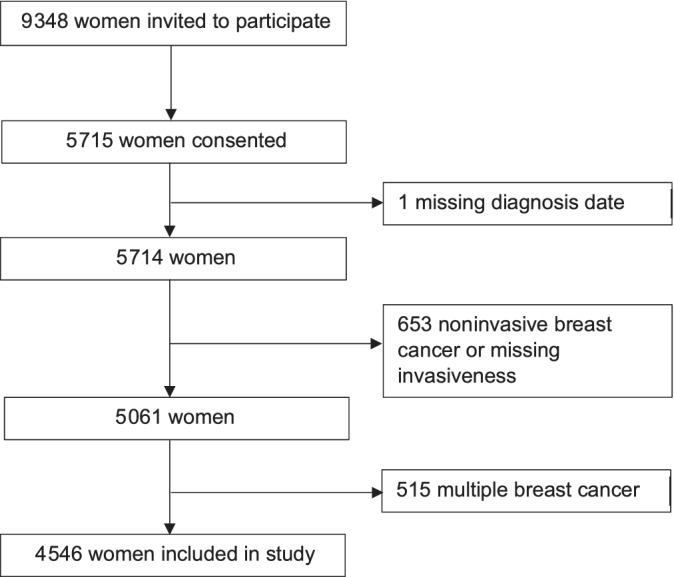
Flow chart describing participants in the study

Women aged less than 80 years diagnosed with breast cancer from 2001 to 2008 and recorded in the Stockholm‐Gotland Regional Breast Cancer quality register (n = 9348) were sent invitations to participate in the LIBRO‐1 population cohort study. A total of 5715 women (61%) consented to participate—they provided blood, answered detailed questionnaire on lifestyle including hormonal and reproductive factors and consented to retrieval of mammography images. Detailed information on the cohort has been published previously.^14^, ^15^, ^16^, ^17^, ^18^, ^19^ From these 5715 women, 1169 were excluded for the following reasons; one woman was excluded due to missing diagnosis date, 653 women had noninvasive breast cancer or missing invasiveness, and 515 women had multiple (including contralateral) breast cancer. This left 4546 women in our study. The flow chart in Figure 1 describes this selection. All study participants gave informed consent and the study was approved by the ethical committee at Karolinska Institutet.

### 2.2 Mammographic sources

Mammograms, both analogue and digital, were retrieved from Departments of Radiology and information on mammography screening history were retrieved from the Stockholm‐Gotland Regional Cancer Center mammography screening database.^16^, ^24^ Mammographic features were evaluated using measures that had the most clinical relevance as described in detail later.

### 2.3 Microcalcification clusters

We used a method developed in our group for the detection of microcalcification clusters that can be applied on different digital systems and vendors, enabling incorporation of both analogue and digital images for large population studies.^25^, ^26^ This method comprises the following steps: (a) image preprocessing, primarily involving denoising, quality improvement and enhancement of small objects, (b) identification of microcalcification candidates, (c) filtering out noise (keeping only objects with shapes, sizes and appearances similar to microcalcifications) and grouping microcalcifications into clusters.^25^ Two microcalcifications are defined to be in the same cluster if they are less than 4.1 mm apart and there has to be at least four microcalcifications to form a cluster.^25^ This threshold was defined based on our experiment during the development of our algorithm in our earlier manuscript.^25^ It is also similar to the threshold value used by the commercial software iCAD, to which we compared our results in our earlier manuscript.^25^ For an example of microcalcification cluster detection in digital image, see figure 2 in previously published paper.^25^ In our study, we evaluated the presence of microcalcification clusters on the cancerous breast and contralateral unaffected side using mediolateral‐oblique (MLO) images closest to diagnosis, defined as 3 years prediagnosis to 3 months postdiagnosis.

### 2.4 Mammographic density and density change

Percentage mammographic density was calculated using the area‐based STRATUS algorithm, which has been developed to analyze a range of image formats, including both analogue and digital images, with automation of density change measurements over time was used.^27^ This method has an in‐built alignment protocol, which reduces nonbiological variation of breast density changes in women.^27^ This measurement was then converted to a categorical variable using cut‐points (2%, 18%, 49%). These cut‐points were taken from previously published work to group the percent density into four breast composition categories in line with clinically relevant Breast Imaging Reporting and Data System (BIRADS; American College of Radiology, Reston, VA) score. This computer‐generated score is termed and abbreviated as cBIRADS.^28^

In the statistical analysis of density, percent density measurements of the contralateral side to the breast cancer were used to ensure that the tumors did not affect image measurements. Examination closest to date of diagnosis was used. After our earlier study that showed increased probability of interval cancer with high mammographic density,^16^ we further investigated the clinical relevance of the associations of mammographic density, evaluated using cBIRADS,^20^ with subtype, tumor characteristics and detection mode.

For density change analysis, relative annual density area change (RDC) on the contralateral breast was computed by taking the difference in area density between two time points (defined as first and last mammography prediagnosis) per baseline density of each women using the equation^29^

$$\mathrm{RDC}=\frac{\left(d_{2}-d_{1}\right)/d_{1}}{t_{2}-t_{1}},$$

where *d*_1_ denotes area density at first mammography *t*_1_, *d*_2_ denotes area density at last mammography *t*_2_, and *t* are times on a yearly unit scale.

### 2.5 Outcome measurements

Tumor and patient characteristics were retrieved from linkages to Stockholm Breast Cancer Quality Registers and the Swedish Cancer Registry. Estrogen receptor (ER) and progesterone receptor (PR) status were determined by immunohistochemistry (IHC) or radioimmunoassay methods and categorized as positive or negative. HER‐2 status was determined by IHC/fluorescence in situ hybridization (FISH) and categorized as positive or negative.

For classification of molecular subtypes, additional data on ER, PR, HER2 and Ki‐67 were obtained from medical and pathology records.^14^ A dataset containing RNA‐sequenced PAM‐50 gene expression was used as training dataset to classify particular molecular subtypes using ER, PR, HER‐2, Ki‐67 and age at diagnosis as inputs using a random forest algorithm.^14^ Full details of the classifier method with robust sensitivity analyses has been published earlier.^14^

Screening history from the mammography‐screening database at the Stockholm‐Gotland Regional Cancer Center was used to determine detection mode.^16^, ^24^ Information on mammography visits and outcomes of individuals attending the population‐based mammography‐screening program in Stockholm County are recorded in the database.^16^, ^24^ From 1989, women aged 50‐69 were being invited for screening every 24 months, and that after mid‐2005 and during the period of our study, women aged 40‐49 were also being invited for screening every 18 months.^16^, ^24^ Detection mode was categorized as interval vs screen‐detected breast cancer. We define (a) interval breast cancer as breast cancer diagnosed after a negative screen but before next scheduled screening or end of a normal screening period, and (b) screen‐detected breast cancer as breast cancer diagnosed with a positive screen during a screening visit.^16^ Individuals who had no mammography screening before a breast cancer diagnosis (ie, not within the recommended screening age or if their previous screening was done more than 18/24 months prior to their breast cancer diagnosis) were excluded from analyses on mode of detection.

### 2.6 Statistical analyses

Multinomial logistic regression was used to individually model molecular subtype, tumor characteristics and mode of detection as a function of each mammographic feature, microcalcification clusters and mammographic density change. Microcalcification clusters were evaluated as a categorical covariate in terms of the presence vs absence of clusters in the cancerous breast and contralateral unaffected breast (separately). In addition, in women with available images from both breasts, we compared the number of microcalcification clusters between the affected and the unaffected sides by taking differences. We categorized the differences into two levels: higher number of microcalcification clusters in the affected breast than the unaffected breast (positive difference), no difference or fewer microcalcification clusters in the affected breast (no positive difference). Mammographic density was evaluated comparing cBIRADS B, C and D vs cBIRADS A. Density area change was evaluated in terms of no density reduction vs density reduction, with reduction defined as negative change in relative annual density area.

For all analyses, models were adjusted for age at diagnosis, body mass index (BMI), hormone replacement therapy (HRT) use and postmenopausal status. For density and density change analyses, models were additionally adjusted for age at mammography. For density change analysis, the model was even adjusted for baseline density. Statistical analyses were performed using R 3.5.1.^30^

## 3 RESULTS

A total of 4546 women with invasive breast cancer satisfying inclusion criteria were included in the study. The majority of women were aged 50 or more (79%) and postmenopausal (80%). For 3361 and 3303 women, images on the affected and unaffected side, respectively, were able to be retrieved from radiology departments for microcalcification analysis. Of which, 3036 women had images on the contralateral side for cBIRADS^20^ density analysis and 1960 women had at least two images on the contralateral side for density change analysis. Detailed baseline characteristics are provided in Table 1.

**TABLE 1:** Characteristics of study population

		Analysis population			
	Full cohort	Women included in analysis of microcalcification clusters in the affected breast	Women included in analysis of microcalcification clusters in the unaffected breast	Women included in analysis of density (cBIRADS)	Women included in analysis of density change
N	4546	3361	3303	3036	1960
Age at diagnosis, years					
≥50	3597 (79.1)	2795 (83.2)	2744 (83.1)	2633 (86.7)	1876 (95.7)
<50	949 (20.9)	566 (16.8)	559 (16.9)	403 (13.3)	84 (4.3)
BMI, kg/m^2^					
≥25	2008 (44.2)	1501 (44.7)	1477 (44.7)	1382 (45.5)	904 (46.1)
<25	2326 (51.2)	1716 (51.1)	1688 (51.1)	1524 (50.2)	986 (50.3)
Missing	212 (4.7)	144 (4.3)	138 (4.2)	130 (4.3)	70 (3.6)
Menopausal status					
Postmenopausal	3634 (79.9)	2815 (83.8)	2766 (83.7)	2647 (87.2)	1881 (96)
Premenopausal	840 (18.5)	506 (15.1)	496 (15)	354 (11.7)	71 (3.6)
Missing	72 (1.6)	40 (1.2)	41 (1.2)	35 (1.2)	8 (0.4)
Hormone replacement therapy use					
Never	2183 (48)	1527 (45.4)	1508 (45.7)	1310 (43.1)	727 (37.1)
Previous	813 (17.9)	641 (19.1)	628 (19)	598 (19.7)	466 (23.8)
Current	756 (16.6)	602 (17.9)	588 (17.8)	564 (18.6)	400 (20.4)
Missing	794 (17.5)	591 (17.6)	579 (17.5)	564 (18.6)	367 (18.7)
Molecular subtype					
Basal	114 (6.3)	72 (5.5)	73 (5.6)	68 (5.6)	33 (4.1)
HER‐2	214 (11.9)	147 (11.2)	145 (11.1)	130 (10.8)	80 (9.9)
Luminal B	174 (9.7)	120 (9.1)	118 (9.1)	98 (8.1)	67 (8.3)
Luminal A	1297 (72.1)	973 (74.2)	966 (74.2)	908 (75.4)	627 (77.7)
ER					
Positive	3656 (84.9)	2731 (86)	2672 (85.6)	2468 (86.1)	1607 (87.3)
Negative	652 (15.1)	446 (14)	448 (14.4)	399 (13.9)	234 (12.7)
PR					
Positive	2985 (70.4)	2243 (71.7)	2198 (71.6)	2017 (71.5)	1280 (70.8)
Negative	1253 (29.6)	884 (28.3)	873 (28.4)	805 (28.5)	529 (29.2)
HER‐2					
Positive	142 (13.4)	87 (12.2)	86 (12.2)	79 (11.4)	52 (11)
Negative	921 (86.6)	626 (87.8)	618 (87.8)	617 (88.6)	419 (89)
Tumor size (mm)					
≥20	1568 (35)	1083 (32.7)	1072 (32.9)	949 (31.7)	549 (28.3)
<20	2906 (65)	2230 (67.3)	2182 (67.1)	2047 (68.3)	1388 (71.7)
Lymph					
Positive	1526 (34.5)	1069 (32.9)	1049 (32.8)	934 (31.8)	563 (30)
Negative	2891 (65.5)	2185 (67.1)	2150 (67.2)	2004 (68.2)	1316 (70)
Grade					
1	571 (19.3)	426 (20.1)	417 (20)	402 (20.8)	287 (22.1)
2	1544 (52.2)	1134 (53.5)	1119 (53.6)	1033 (53.3)	700 (53.8)
3	841 (28.5)	560 (26.4)	552 (26.4)	502 (25.9)	313 (24.1)
Detection mode					
Interval	710 (30)	576 (27.6)	570 (27.7)	537 (26.4)	390 (24.8)
Screen	1656 (70)	1508 (72.4)	1489 (72.3)	1497 (73.6)	1184 (75.2)

*Note:* Values denote numbers (percentage) unless otherwise stated.

### 3.1 Microcalcification clusters

Microcalcification clusters were present in the affected breast for 35% of the women and in the unaffected side for 26% of the women. Results indicated that women with microcalcification clusters in the affected breast were more likely to have a HER‐2 subtype breast cancer OR 1.78 (95% CI 1.24‐2.54) and possibly had a reduced probability of basal subtype OR 0.54 (95% CI 0.30‐0.96) compared to Luminal A subtype (Table 2). In addition, there was a suggestion that women with microcalcification clusters on the affected breast side were less likely to present with interval cancer compared to screen‐detected cancer OR 0.74 (95% CI 0.60‐0.92). In contrast, there were no statistically significant associations involving microcalcification clusters in the unaffected breast, not with subtypes nor with tumor characteristics (Table 2).

**TABLE 2:** Associations of the presence of microcalcification clusters with subtype and tumor characteristics

	Microcalcification clusters in affected breast				Microcalcification clusters in unaffected breast				Difference in microcalcification clusters (affected‐unaffected)			
	OR	95% CI		*P*	OR	95% CI		*P*	OR	95% CI		*P*
**Molecular subtype**												
Luminal A	1.00	—	—	—	1.00	—	—	—	1.00	—	—	—
Luminal B	0.96	0.64	1.43	.845	1.14	0.75	1.72	.539	1.05	0.67	1.65	.823
Basal	**0.54**	**0.30**	**0.96**	**.035**	1.25	0.74	2.10	.406	**0.46**	**0.22**	**0.95**	**.035**
HER‐2	**1.78**	**1.24**	**2.54**	**.002**	1.24	0.85	1.81	.273	**1.77**	**1.20**	**2.60**	**.004**
**ER**												
Positive	1.00	—	—	—	1.00	—	—	—	1.00	—	—	—
Negative	1.20	0.98	1.48	.084	1.04	0.83	1.31	.725	0.82	0.65	1.03	.095
**PR**												
Positive	1.00	—	—	—	1.00	—	—	—	1.00	—	—	—
Negative	1.03	0.88	1.22	.687	1.10	0.92	1.31	.309	0.95	0.79	1.14	.558
**HER‐2**												
Negative	1.00	—	—	—	1.00	—	—	—	1.00	—	—	—
Positive	**2.69**	**1.67**	**4.34**	**.000**	1.27	0.80	2.02	.313	**2.62**	**1.59**	**4.32**	**.000**
**Tumor size (mm)**												
<20	1.00	—	—	—	1.00	—	—	—	1.00	—	—	—
≥20	1.00	0.86	1.17	.974	1.13	0.95	1.33	.160	1.01	0.84	1.20	.951
**Lymph**												
Negative	1.00	—	—	—	1.00	—	—	—	1.00	—	—	—
Positive	0.94	0.81	1.10	.449	1.07	0.91	1.27	.404	0.91	0.76	1.09	.322
**Grade**												
1	1.00	—	—	—	1.00	—	—	—	1.00	—	—	—
2	0.85	0.67	1.07	.174	0.93	0.72	1.19	.560	0.81	0.62	1.05	.114
3	0.94	0.72	1.23	.669	1.07	0.81	1.42	.633	0.96	0.71	1.30	.796
**Detection mode**												
Screen	1.00	—	—	—	1.00	—	—	—	1.00	—	—	—
Interval	**0.74**	**0.60**	**0.92**	**.007**	1.11	0.89	1.38	.362	**0.74**	**0.57**	**0.94**	**.015**

*Note:* Results for affected and unaffected side of breast as well as the difference between them, presented separately.

Bold values indicate findings with *P* < .05.

When using the difference between microcalcifications in the affected and unaffected breasts as an exposure variable, approximately 25% of the women included in our analyses had more microcalcification clusters in the affected side than in the unaffected side (ie, a positive difference). We found a significant positive association between this difference and HER‐2 subtype (OR 1.77; 95% CI 1.2‐2.6) and a negative association with basal subtype (OR 0.46; 95% CI 0.22‐0.95) compared to the Luminal A subtype. Women with positive differences were less likely to be diagnosed as interval cancer compared to screen‐detected cancer (OR 0.74; 95% CI 0.57‐0.94). Detailed results are presented in Table 2.

### 3.2 Mammographic density (cBIRADS)

Based on cBIRADS categories of the contralateral breast, the percentages of women were 9%, 38%, 47% and 6% in categories A, B, C and D, respectively. No significant associations between molecular subtypes and cBIRADS categories were found. Larger tumors were associated with higher density cBIRADS B, C and D, and interval vs screen‐detected cancers were associated with cBIRADS C and D compared to cBIRADS A. Detailed results are presented in Table 3.

**TABLE 3:** Associations of mammographic density with subtype and tumor characteristics. Mammographic density was measured in terms of percent density in terms of cBIRADS categories

	cBIRADS B^a^				cBIRADS C^a^				cBIRADS D^a^			
	OR	95% CI		*P*	OR	95% CI		*P*	OR	95% CI		*P*
**Molecular subtype**												
Luminal A	1.00	—	—	—	1.00	—	—	—	1.00	—	—	—
Luminal B	0.83	0.41	1.68	.595	1.25	0.61	2.59	.543	1.15	0.33	4.03	.830
Basal	0.60	0.24	1.46	.256	0.99	0.40	2.41	.976	0.24	0.03	2.10	.196
HER‐2	1.18	0.58	2.39	.643	0.93	0.45	1.95	.854	2.17	0.83	5.69	.114
**ER**												
Positive	1.00	—	—	—	1.00	—	—	—	1.00	—	—	—
Negative	1.14	0.75	1.73	.542	0.95	0.62	1.46	.809	0.90	0.49	1.66	.738
**PR**												
Positive	1.00	—	—	—	1.00	—	—	—	1.00	—	—	—
Negative	1.26	0.91	1.74	.164	1.17	0.84	1.63	.352	0.80	0.49	1.31	.371
**HER‐2**												
Negative	1.00	—	—	—	1.00	—	—	—	1.00	—	—	—
Positive	1.02	0.50	2.10	.949	0.60	0.27	1.36	.220	1.20	0.37	3.86	.761
**Tumor size (mm)**												
<20	1.00	—	—	—	1.00	—	—	—	1.00	—	—	—
≥20	**1.40**	**1.03**	**1.91**	**.034**	**1.61**	**1.17**	**2.21**	**.003**	**2.27**	**1.46**	**3.51**	**.000**
**Lymph**												
Negative	1.00	—	—	—	1.00	—	—	—	1.00	—	—	—
**Positive**	1.04	0.77	1.41	.803	1.13	0.83	1.54	.443	1.30	0.84	2.01	.233
**Grade**												
1	1.00	—	—	—	1.00	—	—	—	1.00	—	—	—
2	1.01	0.66	1.55	.952	1.02	0.66	1.58	.935	1.25	0.64	2.46	.510
3	1.03	0.63	1.68	.903	0.79	0.48	1.30	.347	0.68	0.31	1.52	.351
**Detection mode**												
Screen	1.00	—	—	—	1.00	—	—	—	1.00	—	—	—
Interval	1.59	1.00	2.54	.050	**2.86**	**1.80**	**4.56**	**.000**	**3.84**	**2.02**	**7.29**	**.000**

a In comparison with cBIRADS A.

*Note:* Bold values indicate findings with *P* < .05.

### 3.3 Mammographic density change

36% of the women considered in this analysis showed no reduction in relative density area. Results suggested that the lack of relative density area reduction over time was associated with Luminal B subtype vs Luminal A subtype OR 1.76 (95% CI 1.04‐2.99) (Table 4). In addition, the lack of density area reduction over time also showed increased odds for interval vs screen‐detected cancers with OR 1.43 (95% CI 1.11‐1.83). However, no significant associations were observed between relative density area reduction and tumor characteristics including tumor size, lymph status and tumor grade (Table 4).

**TABLE 4:** Associations of mammographic relative density area change per year with subtype and tumor characteristics

	No relative density area reduction			
	OR	95%	CI	*P*
**Molecular subtype**				
Luminal A	1.00	—	—	—
Luminal B	**1.76**	**1.04**	**2.99**	**.035**
Basal	0.67	0.29	1.55	.349
HER‐2	1.32	0.80	2.18	.280
**ER**				
Positive	1.00	—	—	—
Negative	0.96	0.72	1.29	.794
**PR**				
Positive	1.00	—	—	—
Negative	1.00	0.81	1.25	.969
**HER‐2**				
Negative	1.00	—	—	—
Positive	1.27	0.69	2.33	.447
**Tumor size (mm)**				
<20	1.00	—	—	—
≥20	1.15	0.93	1.42	.200
**Lymph**				
Negative	1.00	—	—	—
Positive	1.02	0.82	1.25	.887
**Grade**				
1	1.00	—	—	—
2	0.96	0.72	1.28	.774
3	0.91	0.64	1.29	.593
**Detection mode**				
Screen	1.00	—	—	—
Interval	**1.43**	**1.11**	**1.83**	**.006**

*Note:* Density change was measured in terms of no density reduction vs density reduction.

Bold values indicate findings wit *P* < .05.

## 4 DISCUSSION

In our study, we found that mammographic features including microcalcification clusters, cBIRADS density and density change have the potential to be useful predictors for particular invasive breast cancer subtypes and tumor characteristics in the early stages of tumorigenesis. Women with microcalcification clusters in the affected breast were more likely to present with the HER‐2 subtype but potentially less likely to be of basal subtype compared to Luminal A subtype. These women had an elevated probability of being screen‐detected, rather than interval cancers, compared to women without microcalcification clusters. Reassuringly, these associations were only observed on the affected breast side, but not on the contralateral unaffected breast. In addition, high mammographic density measured in terms of clinically relevant cBIRADS showed association with larger tumor size and interval vs screen‐detected cancers. Finally, women with no breast density reduction had increased probability to have interval vs screen‐detected cancers and possibly of Luminal B subtype vs Luminal A subtype tumors.

Microcalcifications in breast tissues are formed through calcium mineralization processes and have been used as indicators of early breast cancer and in particular ductal carcinoma in situ.^10^ Our study is the first to suggest that microcalcification clusters in the affected breast are associated with a reduced probability of having a basal subtype vs Luminal A subtype. It is interesting to note that in studies of unaffected women, microcalcification formation has been shown to be positively associated with breastfeeding,^11^, ^26^ and that among breast cancer patients a history of breastfeeding has been shown to be protective for the basal subtype.^14^, ^26^

In line with previous studies,^12^, ^13^ our study validated the association between HER‐2 subtype and microcalcifications. The exact biological mechanism for this finding remains unknown, although studies have suggested that HER‐2 tumors, which are more aggressive in nature, are more likely to undergo necrosis and fast proliferation, leading to formation of microcalcifications in mammary ducts.^13^, ^31^ Furthermore, our study also suggested a reduced likelihood for interval vs screen‐detected cancers for women with microcalcification clusters. This can partially be explained by the phenomenon that women presenting with microcalcification clusters are more likely to be recalled for additional examinations, and hence, diagnosed with screen‐detected breast cancer.

Consistent with our earlier studies using mammographic density measured on a general numeric/categorical scale,^16^, ^17^ we found significant associations of mammographic density measured using clinically relevant cBIRADS with tumor size and detection mode in our study. This is a finding largely explained by the masking effect, whereby high mammographic density reduces mammographic sensitivity, leading to delayed diagnoses and possibly more advanced tumors.^32^

The association of subtype‐specific tumors with density change over time has, to our knowledge, not been previously studied. Our findings suggested that lack of mammographic density reduction over time was associated with Luminal B vs Luminal A subtype. A general lack of physiological breast density reduction over time might suggest high proliferation rate of breast tissues, which could in turn predispose women to the Luminal B subtype, which is known to be highly proliferative in nature and more aggressive than Luminal A subtype.^16^, ^21^, ^22^, ^23^, ^33^, ^34^, ^35^, ^36^, ^37^ Finally, the association of interval vs screen‐detected cancers with lack of density reduction can be explained by the masking effect, which leads to reduced screening sensitivity and delayed diagnoses.^32^

The strengths of our study include our ability to incorporate both analogue and digital images from different digital systems and vendors, hence enabling a large population analysis of mammographic features in relation to particular breast cancer subtypes and tumor characteristics.^25^ However, one potential limitation is that this method is not currently used in clinical practice and might not fully emulate clinical settings, although earlier study has demonstrated moderate agreement with the performance of computer‐aided diagnosis routinely used in clinic.^25^

Another strength of our study is that we have been able to include within‐person analyses when studying microcalcifications. We have, however, not carried out corresponding analyses for mammographic density (cBIRADS). Prior studies in the literature have reported that mammographic feature asymmetry between breasts predicts individual near‐term breast cancer risk on the next sequential screening mammogram.^28^, ^37^ A recent study showed comparatively higher dense volume and volumetric percent density with time in the cancer‐affected breast compared to the healthy‐breast side.^38^ Therefore, we think that it is problematic to study density in the affected breast, as density in the affected breast would be related to the tumor size, which in turn might be associated with molecular subtype. For a study aimed at testing the hypothesis that density heterogeneity between left and right breasts is associated with molecular subtypes of breast cancer, it would be important to have strong control of the timing of mammograms to ensure that there are no signs of tumors in all included mammograms.

## 5 CONCLUSIONS

Mammographic features (microcalcification clusters, cBIRADS density and density change) could potentially be used as early markers to identify women at increased risk of developing aggressive breast tumors. Current breast cancer risk models identify women who will be at risk for breast cancer,^4^, ^5^, ^6^ but no risk model identifies women at risk for aggressive disease. Future research should evaluate the utility of combining breast cancer risk factors with mammographic features in existing risk models,^4^, ^5^, ^6^ to identify women at risk for developing aggressive breast cancer and in need of supplemental screening.

## CONFLICT OF INTEREST

PST consulted for AstraZeneca and Duke‐NUS. Other authors declare no potential conflicts of interest.

## ETHICS STATEMENT

All study participants gave informed consent and the study was approved by the Regional Ethical Review Board in Stockholm, Sweden (Karolinska Institutet, DNR2009/254‐31/4).

## Data Availability

Datasets generated during and/or analyzed in this study are protected under data protection laws in Sweden and could not be made publicly available. Application for data can be made via the Swedish National Board of Health and Welfare and Statistics Sweden. More information is available from https://bestalladata.socialstyrelsen.se/data-for-forskning/ and http://www.scb.se/Vara-tjanster/bestalla-mikrodata/
